# 1152. Microbiology of Pediatric Neck Infections Based on Age and Anatomic Location

**DOI:** 10.1093/ofid/ofab466.1345

**Published:** 2021-12-04

**Authors:** Joana Dimo, Tracy N Zembles, Glenn Bushee, Michelle L Mitchell

**Affiliations:** 1 Medical College of Wisconsin Affiliated Hospitals/Children’s Wisconsin, Wauwatosa, Wisconsin; 2 Children’s Hospital of Wisconsin, Menomonee Falls, WI

## Abstract

**Background:**

Studies of pediatric neck infections demonstrate an increase in methicillin resistant *Staphylococcus aureus* (MRSA), and predominance of *Staphylococcus aureus* (S. aureus) in infants, and commonly polymicrobial infections. Thus, some providers treat acute neck infections with empiric broad spectrum antibiotics, often with two drugs. Our institution often uses clindamycin plus ampicillin-sulbactam as empiric therapy for hospitalized children with acute neck infection. We aimed to identify the microbiology of acute neck abscesses at our institution to determine if stratifying by age and abscess location would allow for single agent therapy.

Table 1. Causative organism based on anatomic location of neck infection.

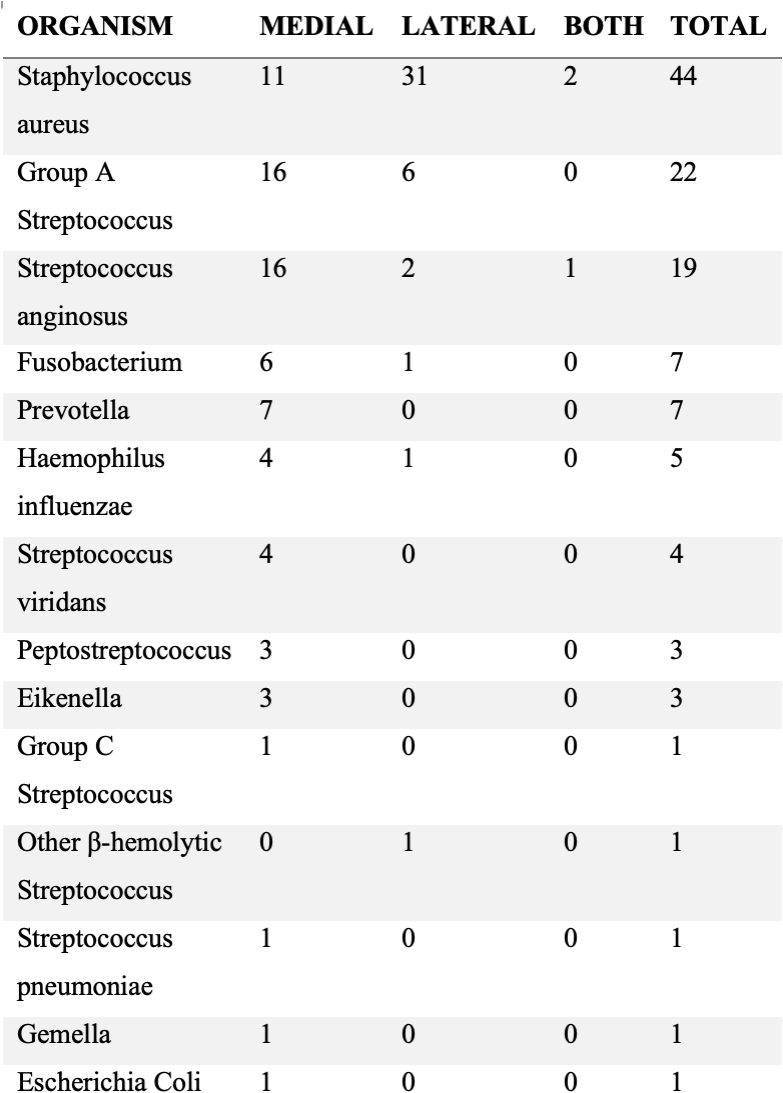

**Methods:**

Diagnosis codes identified patients hospitalized with acute neck infections. Cases with underlying malignancy, cervicofacial malformations, or lymphatic malformations were excluded. Patients with surgical cultures were categorized into two groups based on anatomic location of infection: medial (retropharyngeal, parapharyngeal, and peritonsillar), lateral (other locations), or both. Within each group, causative pathogen(s) were explored and further categorized by age (infants: < 1 year old; non-infants: ≥1 year old).

**Results:**

412 patients were hospitalized for acute neck infection of which 132 had surgical cultures. 110 had growth of one or more pathogens (20 infants, 90 non-infants). 53 infections were located medially, 54 laterally, and 3 had both locations involved. S. aureus was most commonly identified, with lateral infections accounting for the majority (Table 1). 40/44 S. aureus isolates were susceptible to clindamycin. Among medial infections, *Streptococcus Anginosus* and Group A Streptococcus were most common followed by S. aureus (Table 1). 17/20 (85%) positive cultures in infants grew S. aureus with 8/17 (47%) MRSA. No polymicrobial infections were identified in infants. Among non-infants, 0/39 lateral infections had polymicrobial growth but 23/50 (46%) of medial infections did.

**Conclusion:**

Local epidemiology based on anatomic location and patient age suggests a single agent (clindamycin for lateral and penicillin with beta-lactamase inhibitor for medial) may be reasonable for non-infants with uncomplicated neck infections. For infants, coverage of MRSA, regardless of anatomic location, is advisable.

**Disclosures:**

**All Authors**: No reported disclosures

